# A Rare Case of Cronkhite-Canada Syndrome in a Moroccan Female Patient

**DOI:** 10.7759/cureus.79977

**Published:** 2025-03-03

**Authors:** Nadir Miry, Amal Bennani, Ghizlane Kharrasse

**Affiliations:** 1 Department of Pathology, Mohammed VI University Hospital, Oujda, MAR; 2 Faculty of Medicine and Pharmacy of Oujda, Mohammed First University, Oujda, MAR; 3 Department of Gastroenterology and Hepatology, Mohammed VI University Hospital, Oujda, MAR

**Keywords:** case report, cronkhite-canada syndrome, digestive polyps, ectodermal abnormalities, gastrointestinal polyposis, syndromic polyposis

## Abstract

Cronkhite-Canada syndrome (CCS) is a rare nonhereditary disorder characterized by gastrointestinal polyps and ectodermal manifestations, such as alopecia and nail dystrophy. This report highlights the diagnostic and therapeutic challenges of CCS and contributes to the global understanding of the syndrome. A 50-year-old woman presented with persistent anorexia, fatigue, abdominal discomfort, alopecia, and onychodystrophy. Diagnostic evaluation revealed characteristic endoscopic findings of gastrointestinal polyps and histopathological features consistent with CCS. The patient was treated with glucocorticoids, resulting in clinical and nutritional improvement. Nutritional support and immunosuppressive agents may serve as adjunct therapies, but further research is needed to establish definitive guidelines.

This case underscores the importance of thorough endoscopic and histopathological evaluations in rare syndromes like CCS. It also emphasizes the significance of early diagnosis and a multidisciplinary approach to disease management.

## Introduction

Cronkhite-Canada syndrome (CCS), also known as polyposis-pigmentation-alopecia-onychodystrophy syndrome [1], is a rare and non-hereditary disease [2]. It was first described in 1955, and its designation as CCS was conferred by Jarnum and Jensen in 1966 [3]. The prominent clinical manifestations of CCS encompass symptoms associated with gastrointestinal polyps and their severe complications, such as gastrointestinal bleeding and intestinal intussusception [4,5]. Ectodermal symptoms include alopecia, nail dystrophy, and skin pigmentation. This pigmentation can manifest diffusely or focally, often involving the limbs, face, neck, and occasionally the lips [6,7]. Cronkhite-Canada syndrome commonly affects both the upper and lower gastrointestinal tracts while typically sparing the esophagus [8]. The definitive diagnosis of CCS relies on patient history, physical examination, and endoscopic and histopathological findings [9]. From a therapeutic perspective, there is currently no standardized treatment available, as the etiology and pathogenesis of CCS remain unclear. Management relies on glucocorticoids to reduce gastrointestinal inflammation and nutritional support [10].

Our review of the literature, conducted primarily using the PubMed database with keywords such as “Cronkhite-Canada syndrome,” “Cronkhite-Canada polyposis,” and “gastrointestinal polyposis-skin pigmentation-alopecia-fingernail changes syndrome,” did not identify any cases originating from Morocco. Therefore, to the best of our knowledge, this case represents the first documented instance in Morocco. There are over 500 reported cases globally, with 75% originating from Japan [4]. This case report aimed to thoroughly document and analyze this case of CCS in a Moroccan patient. It aimed to provide valuable clinical and histopathological details to enhance understanding and management of this complex condition.

## Case presentation

**Figure 1:**
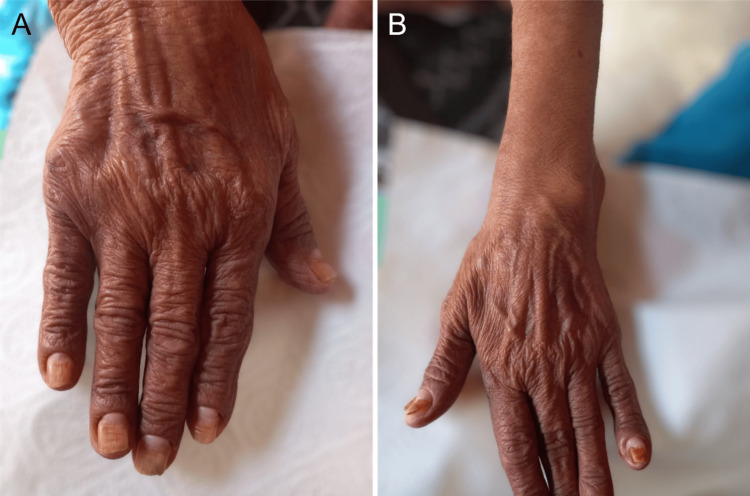
Dermatologic manifestations A: Dystrophic nails; B: Hyperpigmentation on the hand compared to the arm

A 50-year-old female patient presented with complaints of persistent loss of appetite and fatigue. Over the past year, she gradually developed abdominal discomfort, hair loss, nail dystrophy, and skin pigmentation on the hands, all occurring against a backdrop of systemic deterioration. The patient’s symptoms progressively worsened over the past year. Initially, she experienced loss of appetite and fatigue, which later evolved to include abdominal discomfort, hair loss, nail dystrophy, and skin pigmentation on the hands (Figure 1A and Figure 1B). These symptoms were associated with a general decline in her health. The patient had no significant complaints related to her gastrointestinal, respiratory, or urinary systems at the time of presentation.

The patient had no notable surgical, medication, or allergy history. There was no relevant family history of gastrointestinal polyposis or colorectal malignancies, and the patient had no known genetic disorders. Upon admission, the patient exhibited an anemic appearance, with oral mucosal hyperpigmentation and digital nail atrophy. No other significant findings were noted upon physical examination.

Laboratory tests revealed isolated erythrocytosis. Liver and kidney function tests were within normal limits. Screening for *Helicobacter pylori *(*H. pylori*) infection, stool parasitology, and other relevant investigations (such as a complete blood count and inflammatory markers) yielded unremarkable results.

**Figure 2:**
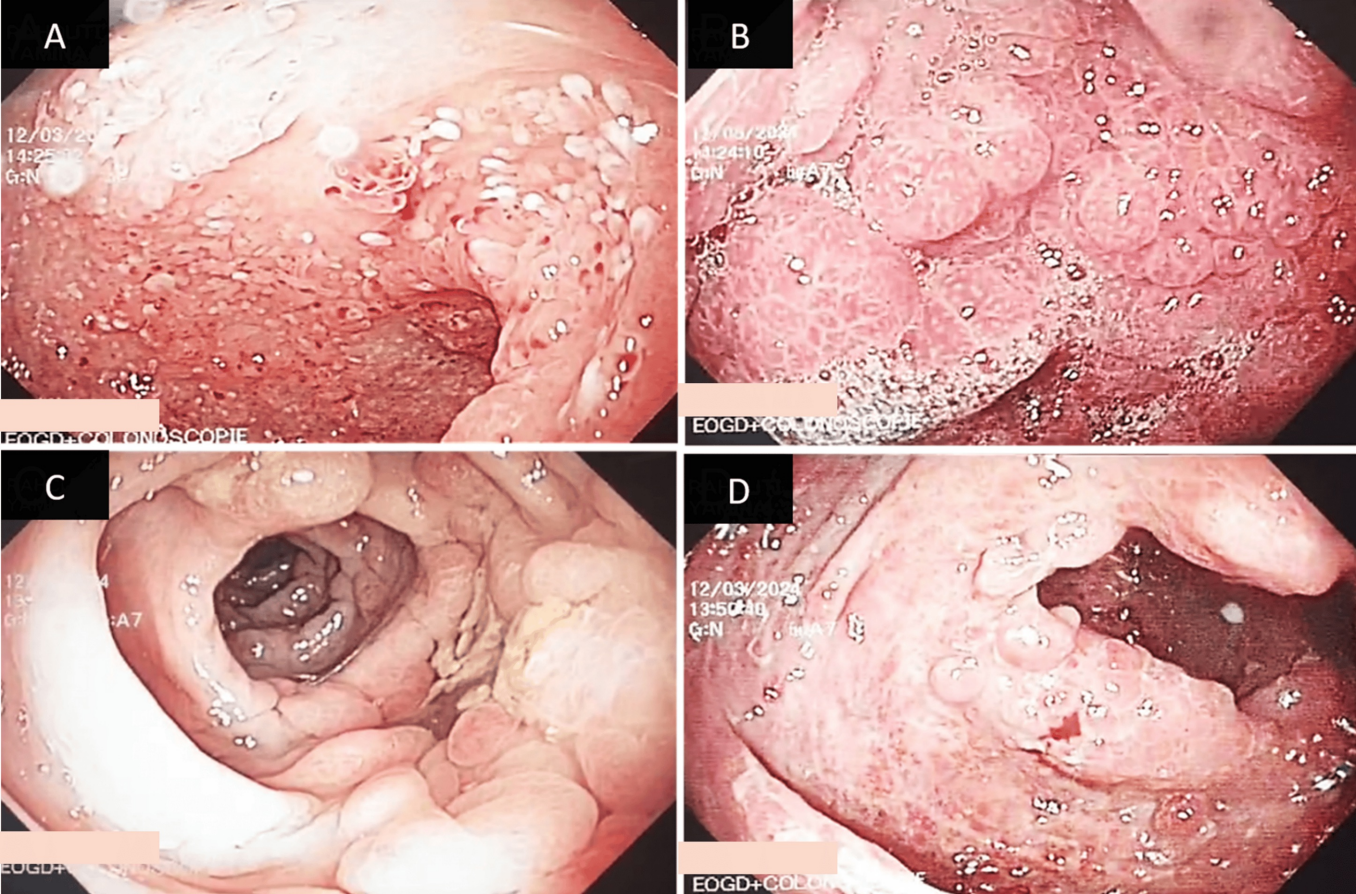
The patient's endoscopic findings A: Multiple polyps and mucosal edema in the stomach; B: Diffuse polypoid lesions with thickened mucosal folds in the duodenum; C and D: Numerous sessile polyps with scattered erythematous areas in the left colon (C) and the cecum (D).

Gastroduodenoscopy revealed swelling and nodular formations resembling polyps in the fundus, gastric antrum, and duodenal mucosa (Figure 2A and Figure 2B). A colonoscopy showed numerous polypoid lesions scattered across swollen mucosa at the ileocecal and colonic levels. The mucosal surface of these lesions exhibited a granular and congested appearance, with the largest lesion measuring 1 cm (Figure 2C and Figure 2D).

**Figure 3:**
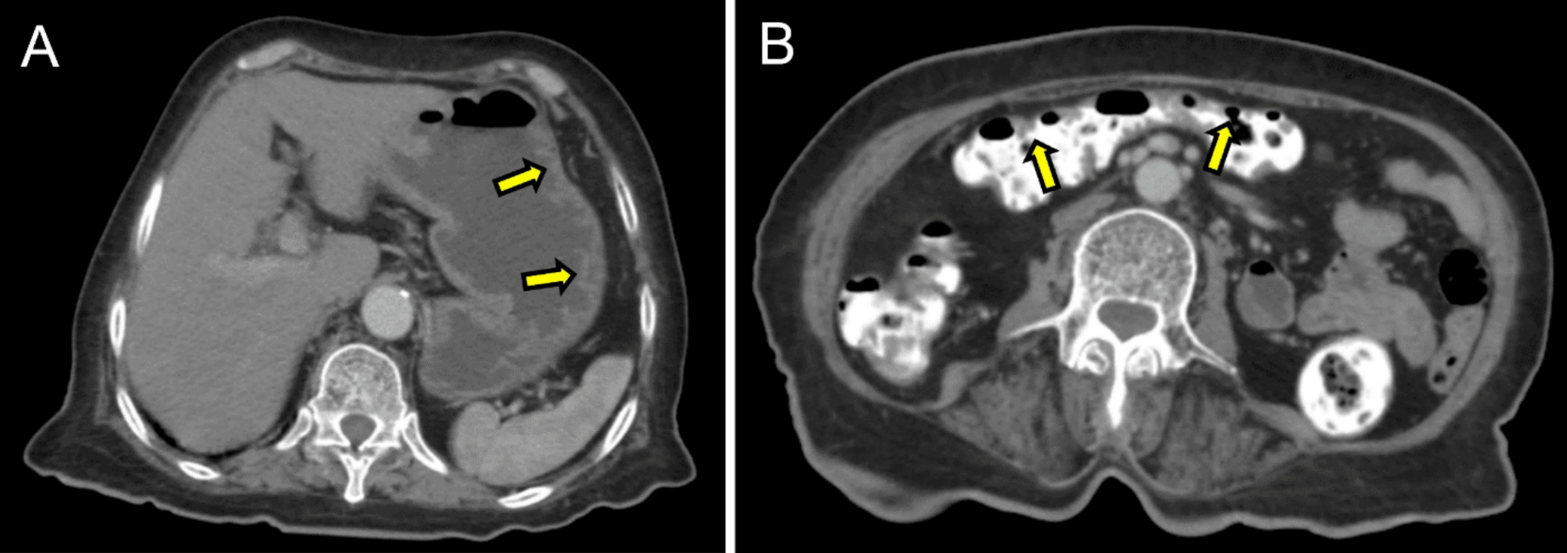
Abdominal axial CT Irregular thickening of the gastric wall (arrows) (A) and the colon (arrows) (B) are noted.

An abdominal CT revealed irregular thickening of the mucosal linings in several gastrointestinal regions, including the antropyloric, duodenal, and ileocolonic areas (Figure 3A and Figure 3B).

**Figure 4:**
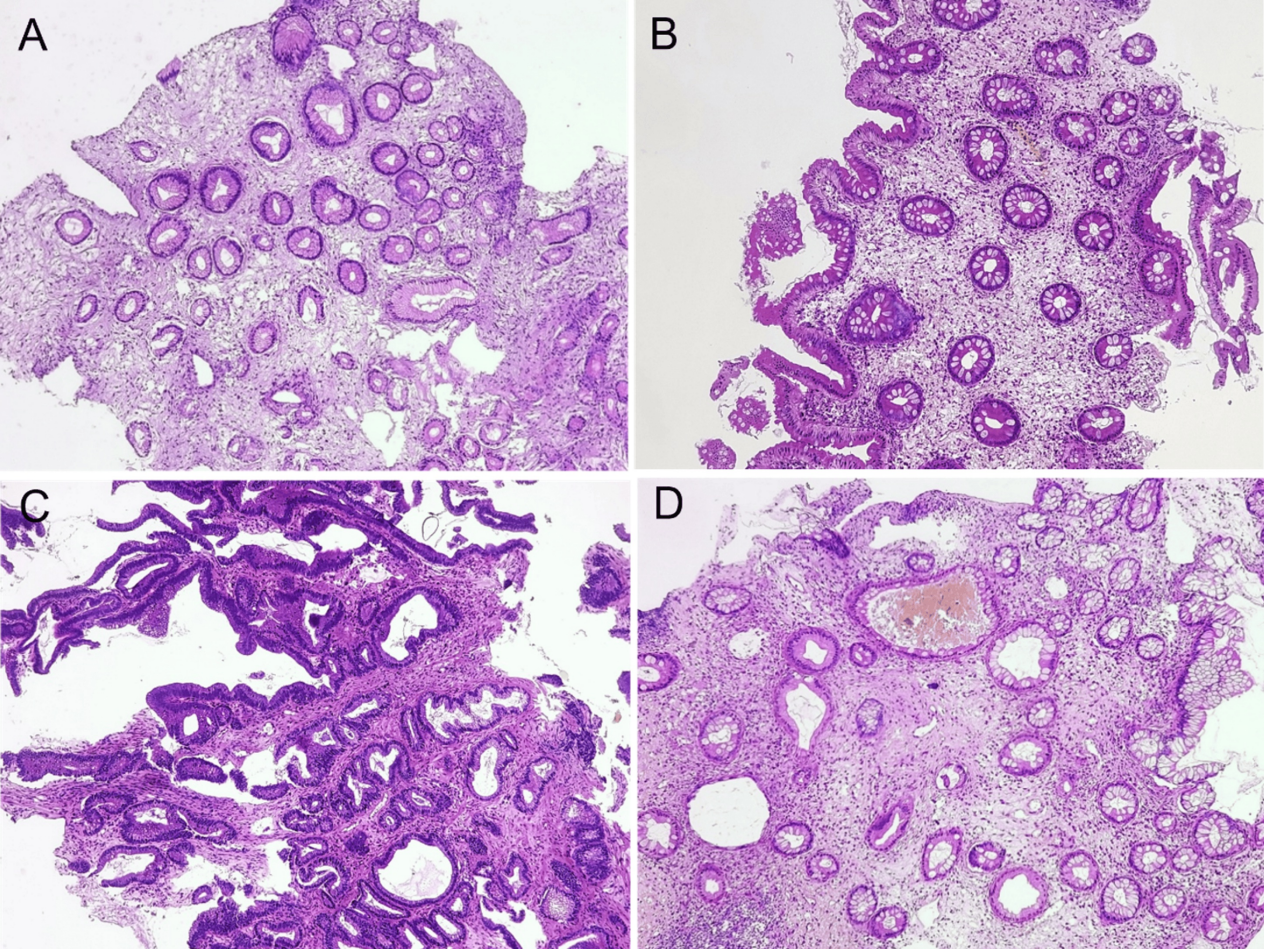
Pathology examination of digestive biopsies A: The stomach biopsy showed foveolar hyperplasia with cystically dilated glands. The lamina propria exhibited edema and infiltration by polymorphous inflammatory cells (×200, hematoxylin and eosin). B: The duodenal biopsy displayed atrophic villi, lamina propria edema, glandular cystic dilation, and inflammatory cell infiltration (×200, hematoxylin and eosin). C: Colonic polyps characterized by dilated and tortuous crypts containing mucin (×200, hematoxylin and eosin). D: The intervening non-polypoid mucosa showed an edematous lamina propria, cystically dilated crypts, and inflammatory cell infiltration (×200, hematoxylin and eosin).

The stomach biopsy revealed foveolar hyperplasia with cystically dilated glands, along with lamina propria edema and polymorphous inflammatory cell infiltration. The duodenal biopsy showed atrophic villi, lamina propria edema, glandular cystic dilatation, and inflammatory cell infiltration. Colonic polyps exhibited dilated and tortuous crypts containing mucin, while the intervening non-polypoid mucosa demonstrated edematous lamina propria, cystically dilated crypts, and inflammatory cell infiltration (Figure 4).

A multidisciplinary team comprising gastroenterologists, dermatologists, pathologists, and nutritionists was involved in evaluating this case to address gastrointestinal and dermatological findings, as well as nutritional concerns.

The clinical presentation, endoscopic findings, and histopathological examination suggested a diagnosis of CCS based on the constellation of symptoms and polyposis. The patient, identified as nutritionally compromised based on her body mass index and nutritional risk index, received parenteral nutrition. She was also started on a 10-day course of corticosteroids (1 mg/kg/day). Following corticosteroid treatment, the patient continued treatment with Modulen® IBD for three months. This regimen provided both nutritional recovery and immunomodulation via transforming growth factor-beta (β), which effectively reduced inflammation and supported clinical improvement. Following treatment, the patient showed clinical improvement, including fewer complaints, reduced diarrhea, improved appetite, and weight gain. The treatment continued to support nutritional recovery and sustained clinical improvement.

## Discussion

Cronkhite-Canada syndrome is an extremely rare clinical condition characterized by epithelial disturbances in both the gastrointestinal tract and the skin. Cronkhite-Canada syndrome has an incidence of approximately one in 1,000,000 and has increased in recent years. Unlike most polyposis syndromes, CCS typically manifests in middle to late adulthood, with an average onset age of 59 years. There is no difference in disease incidence between male and female patients in Europe or the United States [11]. The etiology and pathogenesis of CCS remain largely unknown, with several factors potentially involved. Patients with CCS often exhibit positive antinuclear antibodies and high IgG4 levels, suggesting an autoimmune component. This is supported by the infiltration of polyps by IgG4-positive plasma cells and a favorable response to immunosuppressive treatments. Additionally, a correlation between CCS and *H. pylori* infection has been observed, with some patients showing symptom improvement following *H. pylori* treatment [12-14].

Cronkhite-Canada syndrome is also associated with other autoimmune diseases such as hypothyroidism, membranoproliferative nephropathy, systemic lupus erythematosus, and scleroderma [15]. Mental and physical stress, along with allergic reactions to medications or substances like hair dye, have been suggested as possible triggers [16]. The discontinuation of allergy-inducing agents has been found to reduce IgE concentrations and eosinophil infiltration, improving clinical symptoms. Genetic mutations, such as those in the PRKDC gene, may also play a role in CCS development. Genetic analysis of a mother and her child with CCS identified a specific mutation (C.3921-3925delAAAAG) in the APC gene, indicating a potential genetic factor despite the sporadic nature of CCS [17]. These observations suggest a complex interaction between immune abnormalities, bacterial infections, allergic reactions, and genetic mutations, underscoring the need for further research to elucidate the underlying mechanisms of this complex disease [17].

Cronkhite-Canada syndrome presents a broad spectrum of clinical manifestations impacting both the gastrointestinal tract and the skin. Gastrointestinal symptoms include persistent abdominal pain, chronic diarrhea, nausea, acid regurgitation, loss of appetite, weight loss, and taste disorders, all of which significantly affect patients' quality of life [18]. Cutaneous symptoms feature abnormal skin pigmentation, alopecia affecting the scalp, eyebrows, and eyelashes, and nail abnormalities such as thin, flexible, triangular patches, and atrophy. These skin manifestations are often the first clinical signs and are crucial for diagnosing the disease [19].

The endoscopic features of CCS reveal significant involvement of the gastrointestinal tract, particularly in the small intestine and colon. This includes multiple polyps or nodular elevations with diffuse congestion and edema in the gastric, duodenal, and colonic mucosa, resembling lymphatic dilation changes in the duodenum or terminal ileum. Despite this characteristic presentation, atypical endoscopic manifestations are also observed in some patients, contributing to a higher rate of misdiagnosis or missed diagnosis due to the rarity of the disease. Furthermore, CCS can affect various parts of the gastrointestinal tract, including the esophagus, although the relationship between CCS and esophageal lesions remains unconfirmed.

Endoscopic examinations have revealed the presence of sessile polyps with normal or abnormal mucosa in the stomach, with gastric polyps being most common in the antrum. The severity of lesions tends to increase from the gastric body to the antrum, suggesting a potential association with *H. pylori* infection. Additionally, while polyps may be absent in the stomach, findings such as gastroduodenal inflammation and numerous polyps in the colon are frequently observed. These findings underscore the importance of thorough endoscopic evaluation in diagnosing and managing CCS, facilitating a better understanding of its clinical and pathological features [18,20].

Histopathological examinations of polyp biopsies from patients with CCS reveal a variety of distinct features. The polyps seen are often similar to juvenile, adenomatous, or inflammatory polyps but show stromal and lamina propria changes marked by edema, eosinophilic inflammation, cystic dilatation, and distorted glands with inflammatory infiltration [21]. Unlike juvenile polyposis polyps, CCS polyps are generally sessile with inflammatory cell infiltration and marked edema in the lamina propria. These polyps also show mucosal abnormalities, and although CCS is associated with a high rate of gastrointestinal and colorectal cancers, it remains unclear whether CCS is precancerous or related to the progression of conventional adenoma-carcinoma sequences [19]. Histopathological types of polyps in CCS include inflammatory, non-neoplastic, retention polyps, as well as serrated adenomas and hyperplastic polyps [16].

The incidence of gastrointestinal cancer in patients with CCS is up to 15% and is mainly observed in the colon and less frequently in the stomach. Malignant lesions may arise from pre-existing polyps or occur independently. One study showed that serrated adenomas, which are likely to become malignant, were found in 40% of cases of CCS with colon cancer [15]. These serrated adenomas and hyperplastic polyps are sessile with elongated, dilated, saw-like crypts but do not show significant nuclear dysplasia, which can lead to confusion between the two types on histopathology. In addition to polyps, pathological changes in CCS include inflammatory cellular infiltration dominated by eosinophils, gland/crypt changes with cystic dilatation, edema of the lamina propria, and duodenal villous atrophy. Infiltration of IgG4-positive plasma cells may also occur in CCS polyps, highlighting the complexity of the histopathological manifestations of this rare disease [20].

The differential diagnosis of CCS includes several conditions, such as Ménétrier’s disease, which presents with gastric hypertrophy and protein-losing enteropathy but lacks the systemic features of CCS, such as alopecia and nail changes. Familial adenomatous polyposis and juvenile polyposis are differentiated by specific genetic mutations and distinct histopathological findings. Cowden’s syndrome and Peutz-Jeghers syndrome are characterized by mucocutaneous lesions not seen in CCS. Inflammatory bowel disease may share gastrointestinal symptoms but lacks the systemic manifestations of CCS. Whipple’s disease and lymphomas can be distinguished by microbiological and histological findings. In cases with atypical symptoms and eosinophilic infiltration, additional considerations include eosinophilic gastroenteritis, celiac disease, vasculitis, and parasitic infections [20-22].

Treatment modalities for CCS are diverse, encompassing a variety of medical and surgical interventions. Currently, glucocorticoids, such as prednisone, serve as the cornerstone of treatment, aiming to mitigate gastrointestinal inflammation and suppress autoimmune reactions [20,23]. Alongside glucocorticoids, nutritional support and other immunosuppressive agents like tacrolimus may be utilized to alleviate symptoms [23]. Addressing complications such as severe exudative enteropathy and persistent bleeding may necessitate surgical intervention [20]. Nevertheless, consensus on the optimal management of CCS remains elusive, underscoring the imperative need for further research to establish definitive guidelines and enhance therapeutic efficacy. Nutritional support constitutes a vital component of treatment, encompassing protein-rich diets and supplementation with fluids and electrolytes to facilitate recovery [19]. In our case, the use of Modulen® IBD had a dual effect: it reduced inflammation in the digestive tract and ensured rapid healing of the digestive mucosa. Despite the promising outcomes associated with these approaches, additional investigations are warranted to elucidate their efficacy and specific mechanisms of action within the context of CCS.

## Conclusions

Cronkhite-Canada syndrome poses diagnostic and therapeutic challenges due to its rarity and diverse clinical manifestations. While diagnostic approaches involve a multidisciplinary evaluation, treatment primarily revolves around managing gastrointestinal inflammation and associated complications, often utilizing glucocorticoids and supportive measures. To the best of our knowledge, this case represents the first documented instance of CCS in Morocco, emphasizing the diagnostic and therapeutic challenges posed by this rare condition. It highlights the importance of a multidisciplinary approach and underscores the need for further research to establish standardized treatment guidelines for CCS.
